# Sudden Death in Congenital Heart Disease: The Role of the Autopsy in Determining the Actual Cause

**DOI:** 10.3390/jcdd7040058

**Published:** 2020-12-16

**Authors:** Mary N. Sheppard

**Affiliations:** CRY Unit of Cardiovascular Pathology, Molecular and Clinical Sciences Research Institute, St. George’s Medical School, St. George’s University of London, London SW17 0RE, UK; m.sheppard@sgul.ac.uk

**Keywords:** autopsy, cardiac arrhythmia, congenital heart disease, cardiac fibrosis, cardiac histology, cardiac surgery, surgical complications, pulmonary hypertension, sudden cardiac death

## Abstract

Congenital heart defects (CHDs) have undergone a large change in epidemiology due to prenatal screening and improved outcomes with surgery and percutaneous procedures. In patients with complex CHD there is an increased risk of sudden cardiac death (SCD) and up to 11% of all SCDs in the young occur in people with CHD. It is essential for clinicians to be aware of the risk factors, and for all patients to be followed up in specialised centres. When an SCD occurs, it is important that an autopsy is done and for the pathologist to have an in-depth knowledge of the particular defect and the corrective surgical techniques employed, as well as any complications due to these procedures. Both pathologist and cardiologist should work closely together to explain the cause of death to the family. A terminal cardiac arrhythmia explains many of the SCD cases, often with underlying cardiac fibrosis due to previous procedures. SCD may also be the first presentation of CHD, so great care is required when examining such cases and referral for a detailed expert opinion is recommended in all CHD-SCD cases.

## 1. Introduction

Congenital heart defects (CHDs) are the most common birth defects, affecting approximately 1% of newborns [1]. In the past 50 years there has been a dramatic increase in the survival of patients with congenital heart disease, and they are more likely to survive into adulthood following corrective surgery [2]. The adult congenital heart diseases population is now exceeding the pediatric congenital heart diseases population and is annually increasing. There are approximately three million patients older than 18 years old with CHD in the United States and Europe. However, corrective surgery does not mean that the heart will return to normal. By the age of 50, adults with CHD are at a greater than 10% risk of cardiac arrhythmias and 4% experience sudden cardiac death (SCD) [3].

SCD is described as a sudden, natural, unexpected death; in witnessed cases, as an acute change in cardiovascular status with time to death being <1 h and, in unwitnessed cases, as a person last seen alive <24 h before being found death. In CHD it is referred to as CHD-SCD.

### Clinical Challenges in CHD-SCD

When dealing with both children and adults with CHD, a major challenge for the cardiologist is risk ratifying the individual patients to the risk of SCD. As this population is growing, the risk also increases. Cardiologists who treat both children and adults with CHD are aware of the need for risk stratification, particularly as this population is increasing with long-term haemodynamic changes increasing the risk of SCD [4]. With increasing experience, electrocardiographic abnormalities are becoming more useful in determining the risk of SCD in this population [5]. Cardiac imaging with ECHO and MRI is increasingly applied—particularly to determine cardiac remodelling and fibrosis which puts the patients at increased risk of SCD [6]. Patients with congenital heart disease develop atrial and ventricular arrhythmias, and implantable defibrillators are used for high risk of SCD [7,8]. Antiarrhythmic drugs are first line treatment, but surgical and catheter-based interventions for structural abnormalities and catheter ablation for recurrent ventricular arrhythmias are more widely applied today. The use of implantable defibrillators (ICDS) for CHD is now widely applied, but risk stratification remains poorly defined in many cases and there is also the risk of inappropriate shocks and infections—so great care is needed in their use, as ICDS are not a panacea and do not prevent SCD [9] (otherwise we might all have them implanted for immortality).

In a recent Danish study, there were 809 SCDs in young people between the ages of 0–35 years between 2000 and 2009, of which 90 (11%) had congenital heart disease. This gives a rate of 4.4 per hundred thousand person years. Out of the 90, 64 had autopsies of which 37 (50%) were not diagnosed prior to death. Most had been diagnosed during life, but the Danish study emphasizes that in total 41% of the SCD-CHD cases were not diagnosed with CHD before their sudden death, which highlights the importance of the autopsy in determining the exact cause of the SCD with CHD being important especially in young patients. The most frequent diagnosis was coarctation of aorta, transposition of the great arteries and univentricular heart. The mode of death was rupture of an aortic aneurysm in 13% of cases, while death was presumed arrhythmic in the remaining 87% of cases [10]. This study emphasises that at autopsy there are usually not specific direct findings that will explain the sudden death and interpretation of the findings is important. One must consider that the congenital lesion is incidental and that the true cause of death is sudden arrhythmic death syndrome [11]. In this situation the possibility of electrical abnormalities called ion channelopathies must be considered. These can occur in children as well as adults and they are genetic diseases with mutations in ion channels including sodium, potassium and calcium. In a recent study from our centre of 302 cases of sudden arrhythmogenic deaths syndrome (SADS)with a mean age of 24 years, the main aetiologies were catecholamine polymorphic ventricular tachycardia (CPVT), which is common in children, and the long Q-T syndrome [12]. The taking of genetic material at autopsy is important in establishing the mutations responsible for these electrical abnormalities, and this is known as the molecular autopsy. At present the genetic yield is low at approximately 13% but will increase as more mutations are discovered. We have shown that over half of sudden deaths in young people are due to SADS so it is an important cause of death in young people [13]. It is also important to get a specialist opinion from an expert cardiac pathologist in establishing the cause of death in children when a cardiac cause is being considered, be it congenital heart disease, cardiomyopathies or SADS [14,15].

**Figure 1:**
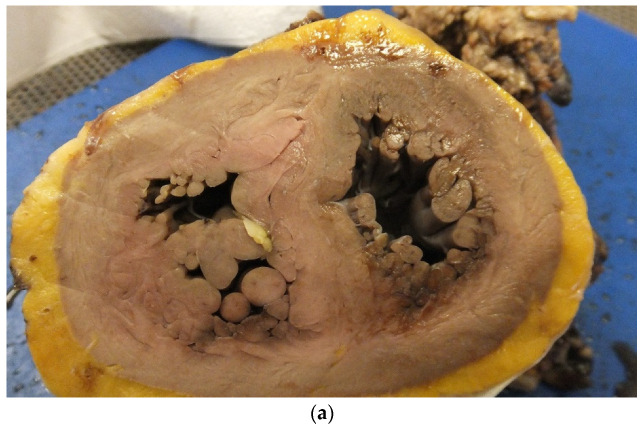

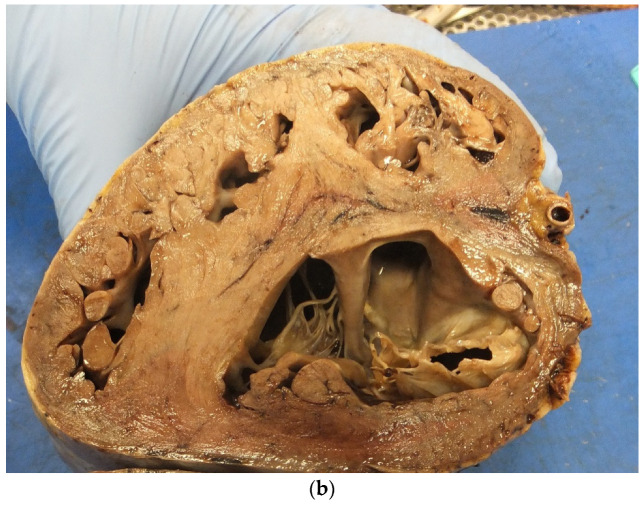
(**a**) A short axis view of a patient with tetralogy of Fallot in which there is marked right ventricular hypertrophy. (**b**) A short axis view of a patient with a thin walled left ventricle and markedly hypertrophied right ventricle in the case of Eisenmenger syndrome with VSD.

**Figure 2:**
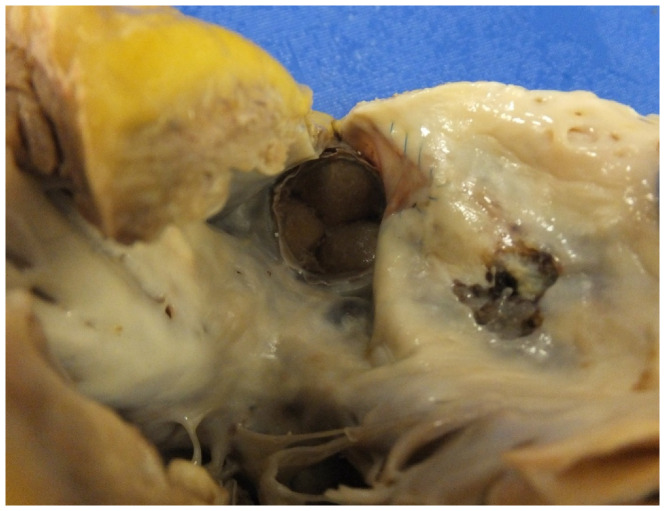
A corrected tetralogy of Fallot in which there is a pericardial patch to open up the right ventricular outflow tract. Note the calcification and ulceration and fibrosis in the areas of patching which can be a substrate for cardiac arrhythmia. Note also the biprosthetic valve in the pulmonary position.

In untreated CHD, there are long-term effects on the myocardium leading to cardiac hypertrophy with replacement fibrosis, and finally cardiac failure with a dilated thin walled ventricle with increased risk of lethal cardiac arrhythmias (Figure 1a,b). In treated CHDs, the interventions may cause further myocardial damage which can also acts as a focus for a cardiac arrhythmia and SCD (Figure 2).

## 2. Value of the Autopsy in CHD

The autopsy is valuable in paediatric cardiology. In an autopsy series in the 1990s on 150 post-operative congenital cases, in 8.5% the autopsy detected unexpected findings which if known prior to death, could have altered therapy [16]. Autopsy can be a very challenging procedure in CHD patients, especially if corrective surgery is carried out with several procedures over a prolonged period of time. The approach and protocol to be used may vary depending on whether the pathologist is facing unoperated cases or previously corrected CHD. Interventions for the same condition have evolved over many decades, as has perioperative myocardial preservations and postoperative care. Careful clinicopathological correlation is required to assist the pathologist in performing the autopsy and reaching a diagnosis regarding the cause of death. Recent guidelines from the European Association of Cardiovascular Pathology describe the most common types of CHDs and include a description of the various types of surgical and percutaneous procedures and major pathological complications. This is a valuable guide to pathologists facing such an autopsy [17]. Small autopsy studies in the forensic area relate to SCD cases with known CHD in the majority but this study also showed that five cases had no diagnosis prior to autopsy, highlighting that the first presentation of CHD can be SCD like in the Danish study [15].

## 3. Surgical Correction and SCD

**Figure 3:**
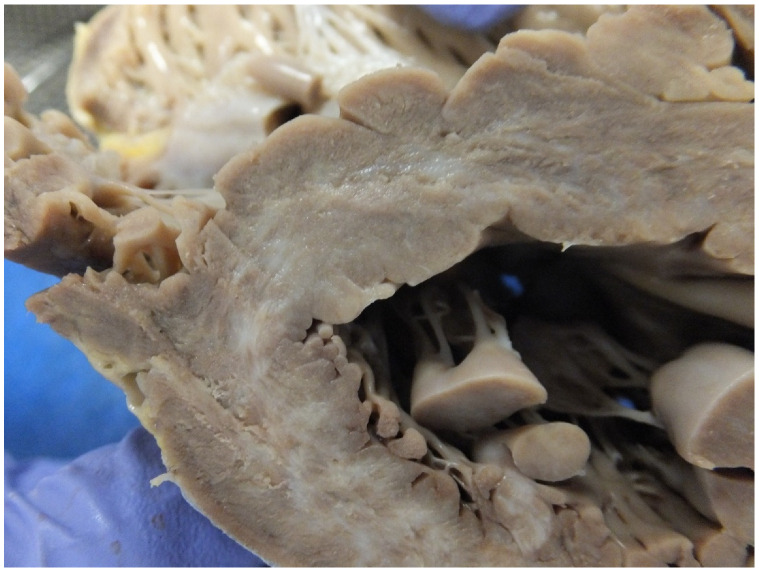
A short axis section of the left ventricle showing replacement pale areas of fibrosis in a case of aortic stenosis.

**Figure 4:**
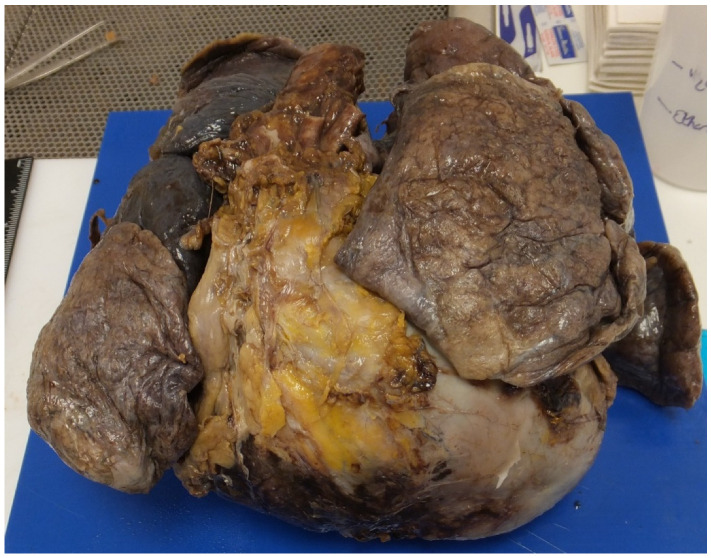
Heart and lungs from the thorax. Note the heart is enlarged and boot shaped extending well beyond the lungs giving the classic appearance of Eisenmenger’s syndrome with biventricular hypertrophy due to a large VSD.

In a study we did in 2006, six cases of surgically corrected complex congenital heart diseases died suddenly, the majority many years after the surgery and there was no indication of clinical deterioration before the sudden death. At autopsy, apart from the surgical corrections and congenital anomalies, there were no specific new findings to explain the sudden death and the death was presumed due to a terminal cardiac arrhythmia, which may be difficult for the family to accept but provides the only explanation in these situations. Why the death occurs at a particular time is unexplained in most cases [13]. In a later study, 21 congenitally malformed hearts were in the same database with and without corrective surgery. High risk lesions were Eisenmenger syndrome, transposition of the great arteries (atrial switch or congenitally corrected) and Fontan circulation. The direct causes of death were attributed to ventricular myocardial fibrosis (11 cases) (Figure 3), pulmonary hypertension (3 cases) and perioperative haemorrhage (2 cases). Again, there were four cases with no explanation for the SCD [18]. Eisenmenger’s syndrome is considered as high/moderate risk for SCD (Figure 4) and is related to age, history of cardiac arrhythmias, heart block, impaired ventricular function and pacemaker insertion and pulmonary hypertension [19,20].

## 4. Pulmonary Hypertension and SCD

**Figure 5:**
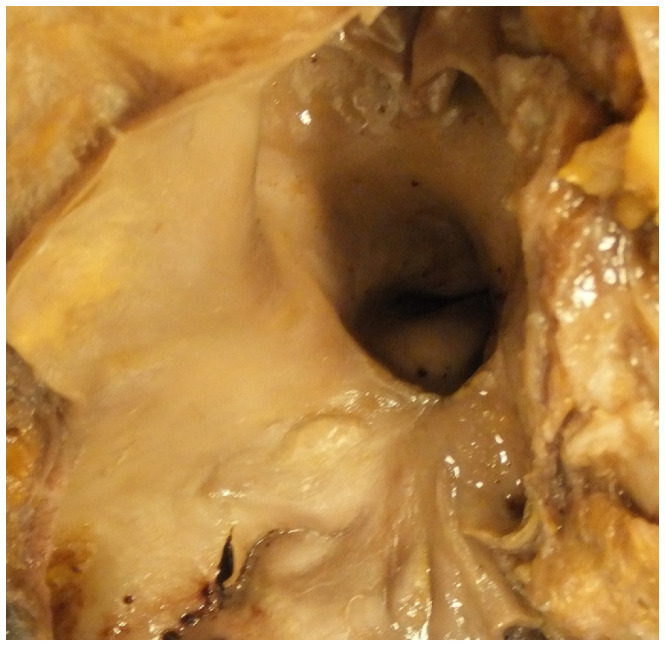
This shows an open pulmonary artery in the case of Eisenmenger syndrome in which there are raised intimal plaques of atheroma present indicating pulmonary hypertension.

**Figure 6:**
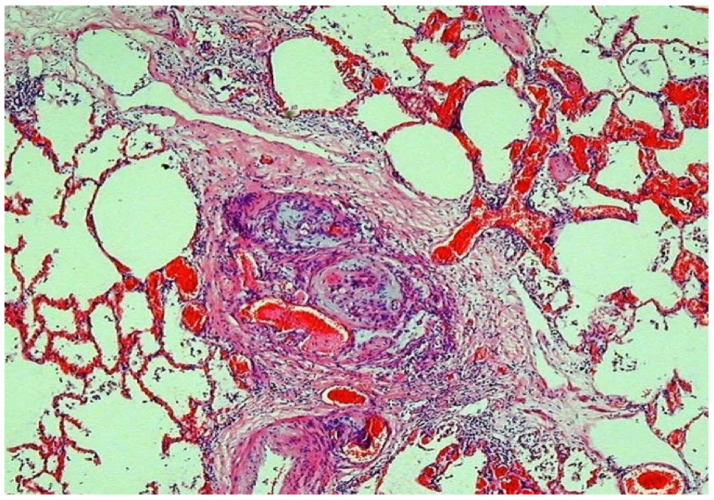
This shows a plexiform lesion in the lung of a case of pulmonary hypertension secondary to congenital heart disease.

Pulmonary hypertension is linked to SCD as already stated. The timing of corrective surgery is important in preventing this, and it can persist despite surgery—especially in older patients. The presence of pulmonary hypertension especially with Eisenmenger syndrome contributes to mortality with right sided heart failure and SCD. Patients with congenital heart disease and pulmonary hypertension are at increased risk of cardiac arrhythmias and arrhythmias are strong predictor of death [21] In a series of 502 Eisenmenger cases, right heart failure is the most common cause of death (29.7%), followed by hemoptysis (18.9%), SCD (16.2%), and pulmonary hypertensive crisis (10.8%) [22]. We did a study of 44 SCD cases with pulmonary hypertensive changes in the lungs at autopsy. In total, 27 cases (61%) had CHD with undiagnosed septal defects and 7 patients were pregnant [23]. This study highlights that many cases can be undiagnosed prior to death and that pregnancy increases the risk of SCD. It is important that clinicians and pathologists be aware of the risk of SCD in asymptomatic patients with pulmonary hypertension, especially in those with congenital heart disease, after cardiac surgery or during pregnancy. Look for the changes with atheroma in the pulmonary arteries at autopsy and extensive sampling of the lungs is essential (Figure 5 and Figure 6). Older procedures such as the Waterston shunt directly between the aorta and pulmonary artery can lead to pulmonary hypertension, in which case death is due to ruptured pulmonary aneurysms in adult life [24] Pulmonary hypertension leads to right ventricular hypertrophy and right-sided arrhythmias and right sided heart failure. In a recent study from China looking at 507 cases of congenital heart disease with pulmonary hypertension, mortality was highest in patients with small defects, less so after correction of the defect and Eisenmenger syndrome, while there were no deaths in patients with pulmonary to systemic shunts. The mode of death in the 37 cases was heart failure (11) haemoptysis (7), pulmonary hypertensive crisis (4) and sudden cardiac death (6) This study emphasises that the causes of the death can be variable but sudden cardiac death is important [22]. It is in these cases that an autopsy needs to be carried out to eliminate direct causes and study the lungs and heart to establish if there is a substrate explaining the terminal cardiac arrhythmia.

## 5. CHD and Pregnancy and SCD

Pregnancy leads to increased demands on the myocardium in CHD and mortality is highest in those with pulmonary arterial hypertension [25]. Two out of 80 of maternal SCDs in our database were due to CHD and both had Eisenmenger’s syndrome with pulmonary hypertension [26].

## 6. Coronary Artery Anomalies

**Figure 7:**
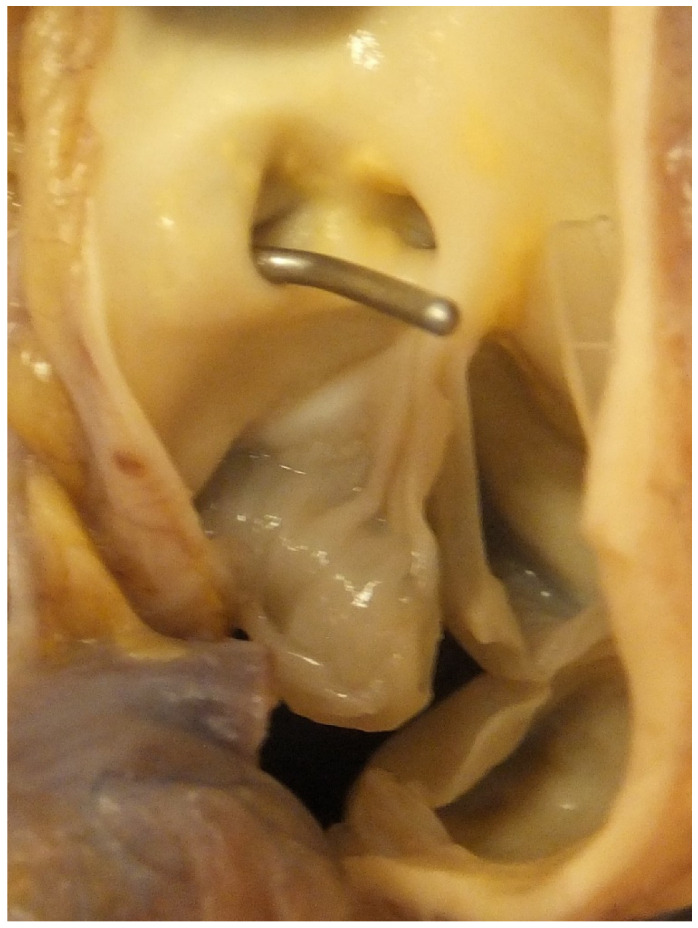
This shows the aortic sinuses and aortic valve. Note a probe is in the ostium of anomalous left coronary artery originating from the right coronary sinus, close to the origin of the right coronary artery.

Congenital coronary artery anomalies are of major significance in clinical cardiology due to their association with myocardial ischaemia and sudden death. Such anomalies are detectable by imaging modalities and their prevalence ranges from 0.21% to 5.79% in angiographic studies. A recent consensus document from the Development, Anatomy and Pathology Working Group of the European Society of Cardiology gives a wide spectrum of coronary artery anomalies [27]. Differentiating benign anomalies from lethal anomalies is obviously important for the pathologist. The proportion of SCDs caused by coronary anomalies has been variably reported ranging from 0% to 4% in the young general population and up to 20% among athletes [28]. Origin from the wrong sinus with interarterial course as well as anomalous origin from the pulmonary artery [29] are the most fatal variants recognized at postmortem of SCDs (Figure 7). It is a rare cause of SCD, but they cause 5% of SCD in sport in our database in the UK [30] Physical exercise is encouraged towards prevention of cardiovascular disease and improvement of ventricular function in congenital heart disease. However, in some congenital heart disease subtypes, especially coronary artery anomalies, physical activity transiently increases the risk of SCD. This is because during exercise there is increased pressure within the great vessels squeezing the anomalous vessels and causing ischaemia. However, ischaemia cannot be detected histologically at autopsy in many cases, and the death is attributed to arrhythmias as a result of the ischaemia [31]. Cardiac arrhythmias causing sudden death is likely in the 50% of cases without overt myocardial damage [31,32]. ICD implantation can act as a secondary prevention of sudden death as reported with ALCAPA [33].

## 7. Valve Disease and SCD

**Figure 8:**
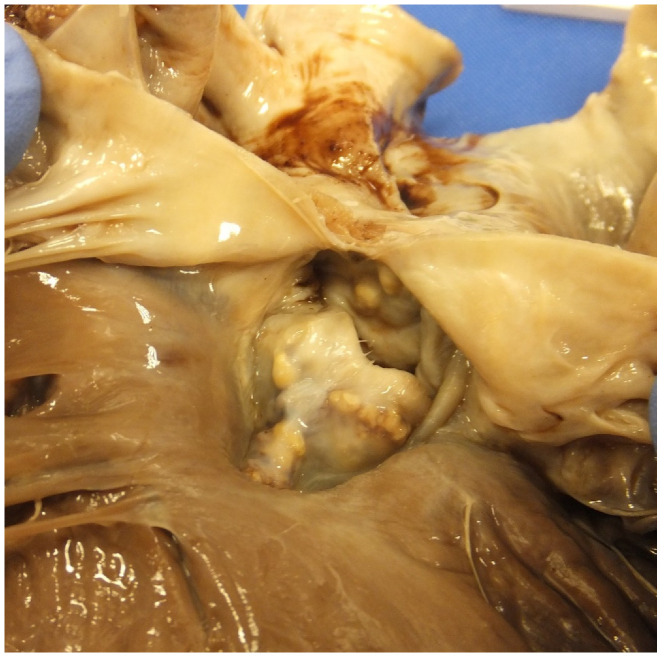
A view of the aorta from the ventricular aspect showing a stenotic calcified bicuspid aortic valve.

Congenital bicuspid valve with calcification and stenosis can lead to SCD (Figure 8) [13]. There are also the complications of endocarditis, regurgitation and aortic dilatation with dissection [34], as well as coarctation. In our recent review of hearts examined at the CRY Centre for Cardiovascular Pathology over a 5-year period, congenital heart valve defects were found in 5% of cases. The most frequent were abnormalities of the heart valves—mitral valve prolapse and bicuspid aortic valve with calcific stenosis. The immediate causes of death are malignant arrhythmias due to significant haemodynamic alterations. Patients with Ebstein’s anomaly of the tricuspid valve often have ventricular pre-excitation which is a risk factor for SCD.

## 8. Endocarditis and CHD and SCD

**Figure 9:**
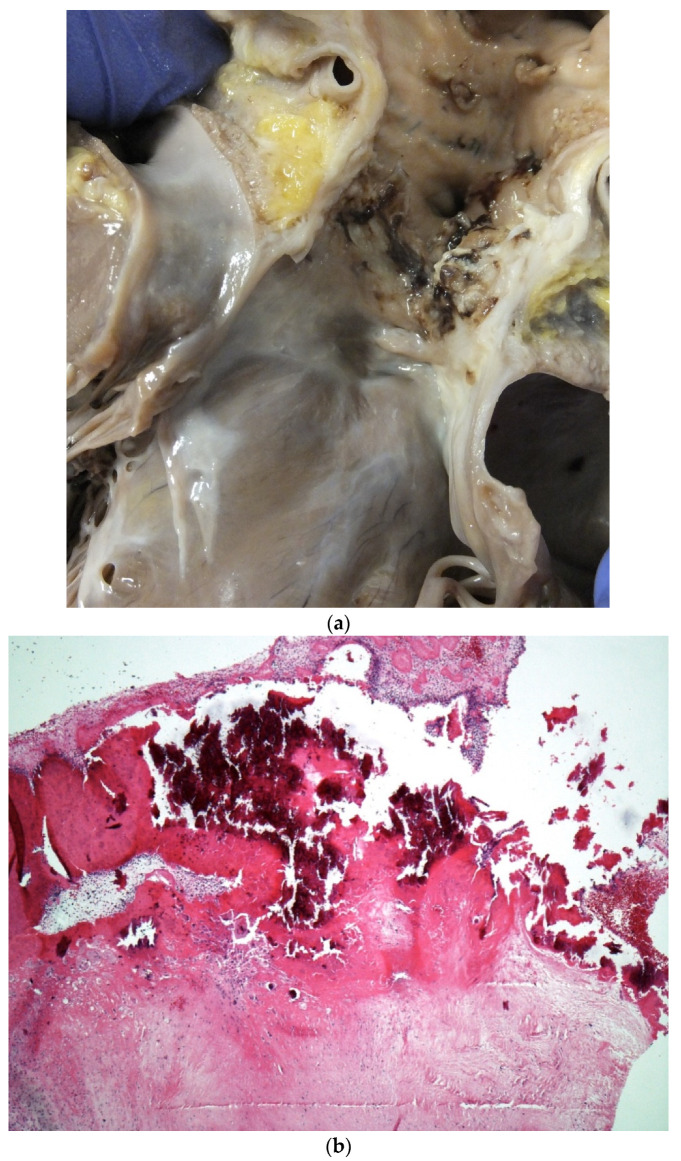
(**a**) A homograft valve in the pulmonary position in a case of repaired tetralogy of Fallot. Note that the valve leaflets have being destroyed by large haemorrhagic vegetations. (**b**) A haematoxylin and eosin-stained section of a valve vegetation with calcification admixed with bacterial colonies as well as acute inflammation and fibrin destroying the valve tissue.

Infective endocarditis (IE) is associated with significant morbidity and mortality in patients with adult congenital heart disease [35] In a recent study we published on 30 cases, of IE and SCD, the main lesion was a bicuspid aortic valve. SCD is due to perforation of the valve leaflets or embolization into the coronary circulation [36] IE can occur in CHD especially with interventions and valve replacements (Figure 9a,b) [37,38].

## 9. Coarctation of the Aorta

This can present a challenge to the pathologist as many cases are missed, as pathologists may attribute left ventricular hypertrophy to perhaps hypertension and they fail to examine the aorta and many of these cases have hypertension. Pathologists may also find a dissection of the aorta and miss that the cause is linked to coarctation with narrowing beyond the left subclavian. Even treated patients are at increased risk of death compared to normal people. A recent study of 834 surgically or endovascular stented patients followed up over a long period showed 38 late deaths and the overall mortality was higher compared to controls. Sixty per cent developed hypertension, which is a risk factor for both aortic rupture and left ventricular hypertrophy [39].

## 10. CHD in Our SCD Database

We have published extensively on SCD, and in a recent analysis of 2653 cases of SCD referred to our pathology unit between 2015 and 2020, 114 (4%) were diagnosed with CHD of which 54% were male and 46% female. The median age was 33 years in both male and female. The most common lesion identified was mitral valve prolapse in 15 cases (13,2%), congenital aortic stenosis in 12 cases (10.5%). anomaly of coronary arteries (9) (7.8%) and pulmonary stenosis 9 (7.89%), tetralogy of Fallot in 8 hearts (7.0%), ventricular septal defect in 6 cases (5.3%), aortopathy with dissection associated either with Williams, Turner or Ehlers–Danlos syndrome in 6 (4.4%) cases and congenitally corrected transposition of the great arteries in 5 cases (4.4%). Isolated large atrial septal defect was found in 4 (3.5%) hearts, in another 3 (2.6%) hearts, atrioventricular septal defect was identified, 3 (2.6%) SCD cases were associated with single-ventricle circulation. Ebstein’s anomaly as well as congenital mitral valve stenosis were identified in 1 heart (1.0%). Fibrosis in the heart was microscopically described in 70 hearts (61%). This study is interesting in that valve lesions are prominent, both mitral valve prolapse and congenital aortic stenosis followed by anomalous coronary arteries while myocardial fibrosis was seen in the majority of cases which could explain the sudden death. It emphasises that sampling of the myocardium will be important at autopsy in any congenital cardiac lesion.

In a recent Spanish study of over 3000 CHD patients, the accumulative sudden cardiac death incidence of 5, 10, 20 years was 0.7, 1.8 and 3.1 respectively. This study emphasises wide differences across specific lesions with high risk cases being Rastelli, severe coronary anomalies, cyanotic heart disease, complex TOF, moderate risk were non-complex TOF, Senning and Mustard cases, Fontan, congenitally corrected transposition of the great vessels (CCTGA), and Ebstein’s anomaly, while coarctation and left heart lesions were low risk. Very low risk cases were left to right shunts and right ventricular outflow tract lesions. This study also used a predictive model indicating that those with a greater than 5% risk as five years follow-up should have an ICD inserted while those with a lower risk of 0.1% should avoid these strategies as ICD can have significant complications [40].

In a recent study from Australia of 2068 patients with CHD 341 died, which is a mortality rate of 11% and this was mainly in complex cases. Non-cardiac causes of death predominated in patients with simple lesions [41]. This highlights that the pathologist should not attribute death simply to the lesion if it is small and insignificant with no remodelling of the heart or lungs. It is important for pathologists to know what are high-risk and low-risk lesions, so that they can make a judgement at autopsy as to whether a congenital lesion found in the heart has actually caused the death of the patient. If a case is only an incidental congenital defect such as a small ASD or VSD, and slight variation of normal coronary anatomy, then the death case should not be attributed to the lesion. Additionally, surgically corrected conditions in which there are no surgical complications, no hypertrophy, dilatation, or fibrosis in the ventricles, should also not have the sudden death attributed to the underlying condition. Coarctation without dilatation and rupture of the aorta or hypertrophy of the left ventricle should also be listed as an incidental finding.

## 11. Summary

SCD is an important cause of mortality in congenital heart disease. Urgent referral for medical treatment of arrhythmias, catheter ablation of arrhythmias and use of implantable cardioverter-defibrillators (ICDs) has improved survival but will not prevent all SCD [7,42,43]. In any case of SCD in CHD it is imperative to carry out an autopsy to clarify the exact cause of death, which helps both the family and the cardiologist in coming to terms with the condition and leading to improvements in monitoring and survival in the future.
